# Case report and literature review: Rare male aggressive angiomyxoma of the scrotum

**DOI:** 10.3389/fsurg.2022.955655

**Published:** 2022-10-31

**Authors:** Yue Chen, YaPing Wei, Hong Chang, ChunKai Yu

**Affiliations:** Department of Pathology, Beijing Shijitan Hospital, Capital Medical University, Beijing, China

**Keywords:** aggressive angiomyxoma, scrotum, local recurrence, male, soft tissue tumor

## Abstract

Aggressive angiomyxoma (AAM) is an uncommon locally infiltrative tumor that frequently occurs in the pelvic soft tissues of female patients; it has a high rate of local recurrence. However, AAM is extremely rare in males. Herein, we present the case of a 70-year-old man with a gradually enlarging painless mass in the scrotum. The patient underwent local excision of the scrotal AAM, with no local relapse after 17 months of follow-up. In addition to the present case, the clinicopathological features of males with AAM reported in literature (to the best of our knowledge) are discussed in this report. The literature review revealed that the gross morphology, clinical process, and histopathology of AAM in males resemble those of AAM in females. In particular, estrogen receptor/progesterone receptor has been shown to be expressed in male patients, which may provide an option for hormone therapy. Moreover, in males, a lower recurrence rate has been observed after surgery to remove the tumor. However, more data are needed to validate this observation. This report emphasizes the importance of considering AAM as the differential diagnosis of myxoid neoplasms in male genital areas.

## Introduction

Aggressive angiomyxoma (AAM), a rare deep soft tissue tumor, occurs predominantly in the pelvis and perineum of women (1). The age of AAM onset in women has previously been reported to range from 6 to 77 years, with the peak incidence during the childbearing years (2). AAM is defined as a benign tumor with no malignant potential, however, AAM has a high probability of local recurrence in the form of local infiltration (3). At present, extensive surgical excision with tumor-free margins is the most commonly available treatment for AAM (2). The occurrence of this type of tumor is very rare in men. To the best of our knowledge, only 85 male AAM cases (including the present one) have been reported in literature (Table 1) since the disease was first described in 1983. In most of these cases, the tumors were detected in the perineal region, inguinal area, and scrotum. AAM is difficult to diagnose without pathological analysis, and misdiagnosis as prostate, testicular, or paratesticular cancer is common (4). Herein, we present the case of AAM arising from the scrotum in a 70-year-old man and describe the diagnosis and treatment procedure along with a literature review on previously reported AAM cases in males. This study was reported in agreement with principles of the CARE guidelines (5).

**Table 1:** The previously reported cases of aggressive angiomyxoma in men.

No.	Reference	Age (y)	Size (cm)	Location	Postoperatively follow	Immunohistochemical profile
1-43	Idrees et al. (6)	1–82	See reference for details			
44	Hatano et al. (7)	59	7.4	Retrovesical area	26 months with no recurrence	CD34 (+), desmin (+), SMA (+), S-100 (–)
45	Wu et al. (8)	40	5.1	Scrotum	12 months with no recurrence	NA
46	Bothig et al. (9)	46	20.0	Perineum	26 months with no recurrence	CD34 (+), desmin (+), S-100 (–), ER (+), PR (+)
47	Pai et al. (10)	48	12.0	Claviculate	6 months with no recurrence	desmin (+), S-100 (–), ER (+), PR (–)
48	Heffernan et al. (11)	54	7.4	Thigh	6 months with no recurrence	CD34 (+), desmin (–), S-100 (–), CD31 (–)
49	Minagawa et al. (12)	37	10.0	Inguinal area	6 months with no recurrence	CD34 (–), desmin (–), S-100 (–), ER (–), PR (–)
50	Sylvester et al. (13)	47	4.0	Larynx	4 years with no recurrence	CD34 (–), desmin (–), SMA (–), S-100 (–)
51	Morag et al. (14)	64	19.0	Scrotum	3 years with no recurrence	CD34 (+), desmin (+), SMA (+), S-100 (–)
52	Plumb et al. (15)	44	26.0	Engrafted kidney	15 months with no recurrence	desmin (+), ER (–), PR (–)
53	Sawada et al. (16)	67	2.0	Prostate	15 months with no recurrence	CD34 (–), desmin (–), S-100 (–), ER (–), PR (+)
54	Bajaj et al. (17)	28	NA	Orbit	4 years with no recurrence	CD34 (+), desmin (+), S-100 (–)
55	Rocco et al. (18)	46	34.0	Scrotum	3 months with local recurrence	NA
56	Mishulin et al. (19)	62	2.5	Orbit	NA	NA
57	Nayal et al. (20)	27	12.0	Axillary region	6 months with no recurrence	CD34 (–), SMA (+), S-100 (+), ER (–)
58	Gaunay et al. (21)	40	5.2	Scrotum	NA	NA
59	Karwacki et al. (22)	81	5.0	Perineum	16 months with no recurrence	CD34 (+), desmin (+), SMA (+), ER (+), PR (+)
60	Saha et al. (23)	17	25.0	Greater omentum	NA	desmin (+)
61	Wang et al. (24)	60	NA	Maxilla	2 months with no recurrence	CD34 (+), desmin (–), S-100 (–), ER (+), PR (–)
62	Caruso et al. (25)	72	23.0	Pararectal	NA	NA
63	Wang et al. (26)	25	6.0	Scrotum	NA	CD34 (+), SMA (–), S-100 (–)
64	Smith et al. (27)	51	11.0	Pelvis	15 months with no recurrence	NA
65	Smith et al. (27)	77	1.2	Knee	NA	NA
66	Draeger et al. (28)	73	NA	Scrotum	No recurrence	CD34 (+), desmin (+), ER (+), PR (+)
67	Ahmed et al. (29)	<1	12.0	Penis	6 months with no recurrence	NA
68	Sharma et al. (30)	53	4.0	Scrotum	NA	NA
69	Gorsi et al. (31)	44	10.5	Engrafted kidney	Dead for pulmonary tuberculosis	CD34 (+), SMA (–), S-100 (+)
70	Damodaran et al. (32)	62	NA	Penis	20 months with no recurrence	NA
71	Ismail et al. (33)	65	16.0	Scrotum	2 years with no recurrence	CD34 (–), desmin (+), S-100 (–), ER (+), PR (–)
72	Aydin et al. (34)	66	15.0	Scrotum	6 months with no recurrence	CD34 (+), desmin (+), S-100 (–), ER (+), PR (+)
73	Umranikar et al. (35)	79	15.7	Perineum	4 years with no recurrence	NA
74	Hsieh et al. (36)	46	20.0	Buttock	NA	CD34 (+), desmin (–), S-100 (–), ER (+), PR (–)
75	Neyaz et al. (37)	53	15.0	Scrotum	12 months with no recurrence	CD34 (+), desmin (+), S-100 (–), ER (–), PR (–)
76	Serao et al. (38)	72	7.7	Paratestis	No recurrence	CD34 (+), desmin (+), PR (+), ER (–)
77	Addesso et al. (39)	56	7.0	Prostate	NA	CD34 (+), desmin (+), S-100 (–), ER (+), PR (+)
78	Kirkilessis et al. (40)	57	11.0	Scrotum	2 years with no recurrence	CD34 (+), desmin (+), S-100 (+), ER (+), PR (+)
79	Liu et al. (41)	62	2.3	Paraureteral area	30 months with no recurrence	CD34 (–), desmin (–), SMA (–), S-100 (+)
80	Majumdar et al. (42)	30	28.0	Jaw	Intraoperative death	NA
81	Celik et al. (43)	55	12.5	Pelvic	16 months with no recurrence	CD34 (–), desmin (+), SMA (–), S-100 (–)
82	Zhu et al. (44)	55	2.0	Prostate	8 months with no recurrence	CD34 (+), desmin (–), S-100 (–)
83	Chen et al. (4)	82	4.7	Paratestis	4 months with no recurrence	CD34 (+), desmin (+), S-100 (–), ER (–), PR (–)
84	Korecka et al. (45)	11	NA	Scrotum	Stable residual mass for 29 months	NA
85	Present case	70	13.0	Scrotum	17 months with no recurrence	CD34 (+), desmin (+), S-100 (–), ER (+), PR (+)

NA, not available.

## Case presentation

### Clinical findings

**Figure 1:**
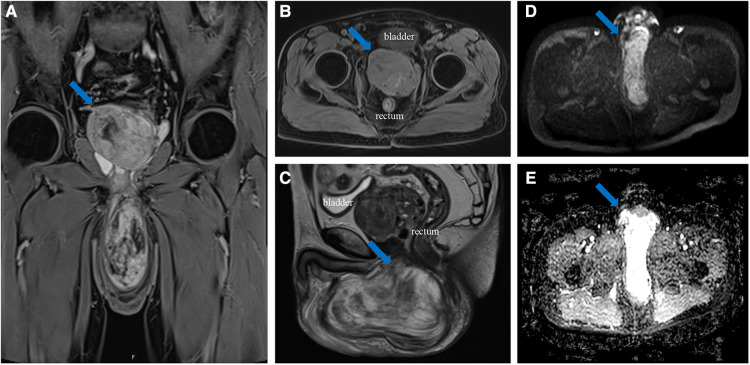
(**A**) coronal (weighted-sequence T1), (**B**) axial (weighted-sequence T1), (**C**) sagittal (weighted-sequence T2), (**D**) axial (DWI), and (**E**) axial (ADC) MRI reveal a well-capsulated mass protruding into the left scrotum in the left pelvis (arrows). ADC_mean_ value = 2.16 × 10^−3^ mm^2^/s. MRI, magnetic resonance imaging; DWI, diffusion-weighted imaging; ADC, apparent diffusion coefficient.

A 70-year-old man presented with a left scrotum mass that had been growing for the past 2 years. The mass was mobile, nonpainful, and had grown progressively larger over time. The patient was not febrile and did not present with frequent urination, urgency, pain, or hematuria. Magnetic resonance imaging (MRI) at another hospital revealed the shadow of a mass in the prostate and left scrotum. Considering the possibility of a malignant lesion, the patient visited our hospital for further diagnosis and treatment. During physical examination, a large, perineal mass approximately 15 cm in size with no obvious blood vessels on the surface was detected. MRI at our hospital revealed a well-capsulated mass protruding into the left scrotum in the left pelvis with a size of 13.2 × 10.0 × 4.3 cm (Figure 1). In addition, an ill-defined mass was detected in the prostate, with a maximum cross-sectional area of 6.6 × 5.5 cm. The patient's total prostate-specific antigen level was 7.572 ng/ml (reference value = 0–4 ng/ml). Excisional biopsy of the scrotal mass was performed after needle biopsy revealed the mass as AAM. As the needle biopsy of the prostate revealed no malignant histological appearance, the patient was not initially treated with surgery.

### Pathological findings

**Figure 2:**
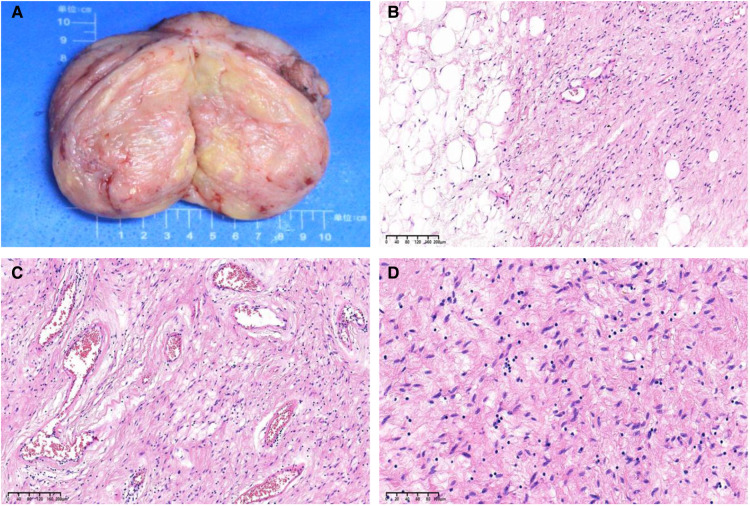
(**A**) A grey–pink or grey–yellow, well-circumscribed, solid mass measuring 13.0 × 7.0 × 5.5 cm. (**B**) Microscopic sections demonstrate a spindle cell tumor entrapping the adipose tissue (hematoxylin and eosin [H&E] stain, ×100 magnification). (**C**) The tumor comprises spindle cells in a loose myxoid matrix containing irregular, variably sized blood vessels and cordlike collagen fibers (H&E stain, ×100 magnification). (**D**) The tumor cells are eosinophilic, small, and spindle shaped, with slightly deep staining and stellate nuclei (H&E stain, ×200 magnification).

**Figure 3:**
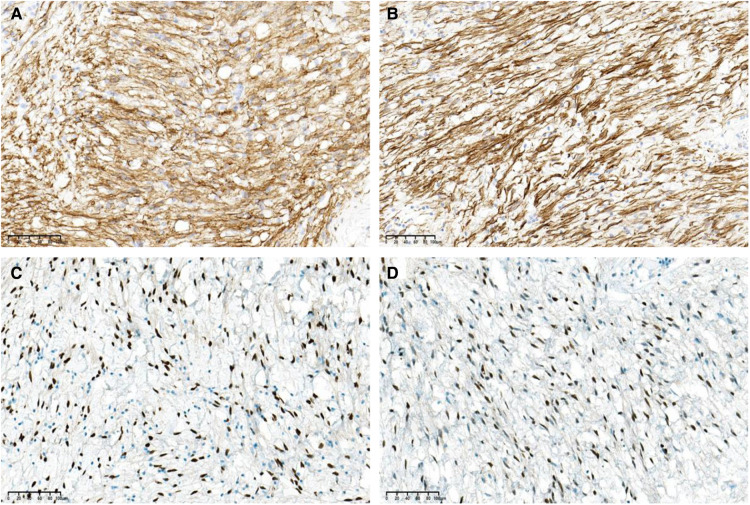
The tumor cells are positive for (**A**) CD34, (**B**) desmin, (**C**) estrogen receptor (ER), and (**D**) progesterone receptor (PR) (immunohistochemical stain, ×200 magnification).

The gross examination of the excisional biopsy revealed a grey–pink or grey–yellow mass measuring 13.0 × 7.0 × 5.5 cm in volume, with a completely smooth capsule on the surface and soft and translucent texture in tissue sections (Figure 2A). Histologically, the tumor comprised spindle cells, myxoid matrix containing thick-walled vessels of varying sizes, and cordlike collagen fibers. The spindle cells were arranged in a wavy or parallel pattern, with no atypia or nuclear division (Figures 2B–D). Immunohistochemical staining revealed that the tumor cells were positive for vimentin, CD34 (Figure 3A), desmin (Figure 3B), estrogen receptor (ER) (Figure 3C), and progesterone receptor (PR) (Figure 3D) but negative for CK, SMA, and S-100. The tumor had a very low Ki-67 proliferation index of 1%. Based on the morphology and the immunological phenotype, the patient was diagnosed with AAM.

The hematoxylin and eosin staining of the prostate needle biopsy did not reveal prostate cancer (Supplementary Figure S1A-B), and immunohistochemical staining showed that the tumor was positive for P63 (Supplementary Figure S1C) and negative for P504S (Supplementary Figure S1D).

### Treatment and follow-up

The patient was treated surgically. First, left arc-shaped incision of the perineum was performed, the perineal skin and muscles were dissected according to the anatomical level and the superficial perineal fascia was dissected. The scrotal mass was located below the urogenital diaphragm (The superior fascia of the urogenital diaphragm is adjacent to the prostate, and the inferior fascia is adjacent to the bulbar corpus cavernosum) and clearly demarcated from the surrounding tissue. The scrotal mass was then surgically removed with clear margins. A needle biopsy of the prostate was also performed intraoperatively. The patient was postoperatively followed-up for 17 months; no recurrence or metastasis has been detected.

## Discussion

AAM was first described as a separate histopathological entity by Steeper and Rosai in 1983. Considering its benign nature, the term “aggressive” was modified to “deep” in the fourth edition of the *World Health Organization Classification of Soft Tissue Tumors* in 2013 (4). In men, only case reports or case series have been reported in literature. The available cases reported in males are described in Table 1 (4, 6–45). These men aged from 9 months to 82 years, with an average age of 49 years. The most common sites were the pelvis and genital areas, particularly the scrotum. Six tumors have been reported in the head and neck area, four in the engrafted kidney or urinary tract, three in the prostate, two in the alimentary tract, two in the lower limbs, one in the axillary region, one in the buttock, and one in the claviculate. Most of these patients were usually asymptomatic, whereas a small number of patients presented with inguinal hernia and testicular tumors. In the present case, the tumor developed in the scrotum and presented as a gradually increasing mass with no typical clinical symptoms.

Most tumors in the reviewed cases were >10 cm in size as AAMs are not easily detected early. The largest tumors found in women and men, respectively, were 60 and 28 cm in size (42, 43). Most tumors are ill defined, making complete resection difficult and resulting in frequent local recurrence. However, few tumors demonstrate partial or complete encapsulation. In most cases, the cut surface was smooth, homogeneous, soft, and gray, with few firm and cystic types (6). In the present case, the tumor was completely encapsulated, with a maximum diameter of 13 cm, and had a smooth, gray, soft cut surface.

Misdiagnosis in AAM is common because it can mimic other diseases, including hydrocele, inguinal hernia, or paratesticular neoplasia (4). Preoperative diagnosis is often difficult and challenging because of the rarity of these tumors and lack of specific imaging features (44). AAM is diagnosed based on the histopathological examination of postoperative specimens. The histopathological features of males with AAM reported in literature are similar to those reported in classical female cases. Microscopically, AAM comprises small-sized spindle cells or stellate cells embedded in a loose myxoid matrix with abundant collagen fibers and variably sized vessels. Blood vessels ranging from capillary-like to thick-walled vessels, which are the most prominent feature of AAM, can be observed (46). In the present case, no evidence of atypical mitotic activity or nuclear atypia was noted. While hypercellularity, cytological atypia, abundant fibrosclerotic stroma, and increased vascularity have been reported in recurrent cases (2), in the present case, the classical morphology was found. Immunohistochemical staining plays a crucial role in the diagnosis of AAM, although there is no specific immunohistochemical marker of AAM. The neoplastic cells of AAM are generally positive for desmin, vimentin, SMA, CD34, ER, and PR but negative for S-100 and CK in female patients (47). Previous studies have shown that ER and PR stains are generally negative in males with AAM compared with females (37). However, in some cases (including the present case), ER and PR may be expressed on AAM tumor, which may provide a hormonal therapeutic option (6). It is worth noting that the relationship of AAM with hormone receptor expression in males has not been well described, and more research is needed in the future. More recently, high mobility group A protein 2 (HMGA2) was revealed as a sensitive but not specific novel marker for AAM diagnosis (48). While nuclear staining can be useful in cases where cytoplasmic staining is nonspecific and of no diagnostic importance (49), the present case was diagnosed without HMGA2 staining considering the typical morphology of the tumor.

AAM should be distinguished from angiomyofibroblastoma, myxoid liposarcoma, myxoma, superficial angiomyxoma, and myxoid neurofibroma. Angiomyofibroblastoma is a benign tumor that has recently been described as histologically similar to AAM, with myofibroblastic cells clustered in abundant myxoid stroma. This tumor contains several areas of hypo- and hypercellular cells, often clustered around blood vessels. Another key histological feature of angiomyofibroblastoma is the presence of multinucleated giant cells (50). Myxoid liposarcoma must be considered as a differential diagnosis when tumor cells infiltrate adipose tissues. It can be easily distinguished from AAM as myxoid liposarcoma is marked with adipocytes set in abundant thin-walled vessels. Myxoma, a benign tumor of the extremities, is characterized by an abundance of myxoid stroma and benign spindle and stellate cells surrounded by small blood vessels (and not blood vessels of different sizes). Superficial angiomyxoma, also known as cutaneous myxoma, usually occurs in the skin of nongenital areas. Histologically, the tumor lacks thick-walled vessels and the myxoid areas can form pools. With respect to the immunophenotype, ER and PR are generally not expressed but S-100 may be expressed in superficial angiomyxoma (51). Myxoid neurofibroma is another AAM-resembling myxoid tumor that commonly occurs in the extremities; however, the clusters of wavy nerve tumor cells are usually strongly S-100 positive.

The surgical removal of AAM with clear margins is the traditional treatment to prevent local recurrence. However, it is not clear whether the recurrence rate is associated with the surgical margin status (52). In women, the recurrence rates of 71%, 85%, and 94% have been observed within the first 3, 5, and 7 years of local excision, respectively (53). As shown in Table 1, 79 of the 85 male patients were followed-up after surgery, and the data revealed only 4 recurrences (4.7%), excluding 2 deaths (one died intraoperatively and one died because of pulmonary tuberculosis). The patients with recurrence were treated with wide excision, and the procedures were performed 9 months to 7 years after first surgery. Possible reasons for the lower local recurrence rate in male patients are sample limitations or lower hormone expression. It is generally known that AAM has no metastatic tendency; however, two female patients (aged 63 and 27 years) showed metastasis to the lung (54, 55). By contrast, as expected, no metastatic cases have been reported in males. Moreover, owing to the low proliferative activity of AAM, the role of radiotherapy and chemotherapy is unclear and limited. In recent years, hormonal therapy has been considered an adjunctive treatment for ER and/or PR positive female patients with primary large mass or local relapse that is not amenable to surgery (56). Unfortunately, it is not clear whether the relapse rate is higher when hormone therapy is discontinued (57). In the present case, only local resection was performed, and although the patient was both ER and PR positive, hormonal therapy was not advised as few data are available on hormonal therapy in male patients with AAM. Long-term follow-up surveillance is required because of the aggression and relapse characteristics of this tumor. At present, the patient has been followed-up for 17 months without any recurrence or metastasis.

## Conclusion

In summary, a rare case of scrotal AAM was reported and previously reported male cases with AAM were summarized. In particular, the review revealed that the clinicopathological characteristics of AAM in men are similar to those of AAM in women, including the expression of ER and PR. This provides an opportunity to treat such male patients with hormone therapy. Moreover, the literature review revealed a low recurrence rate (4.7%) in males after the surgical excision of the tumor; however, more data are needed to confirm this observation. Finally, AAM should be distinguished from myxoid neoplasms in male genital areas.

## Data Availability

The original contributions presented in the study are included in the article/Supplementary Material, further inquiries can be directed to the corresponding author/s.
