# Supplementary figures and images for: Case report and literature review: Rare male aggressive angiomyxoma of the scrotum
Source: Front Surg. 2022 Oct 31;9:955655. doi: 10.3389/fsurg.2022.955655 (PMC9659604; doi:10.3389/fsurg.2022.955655)

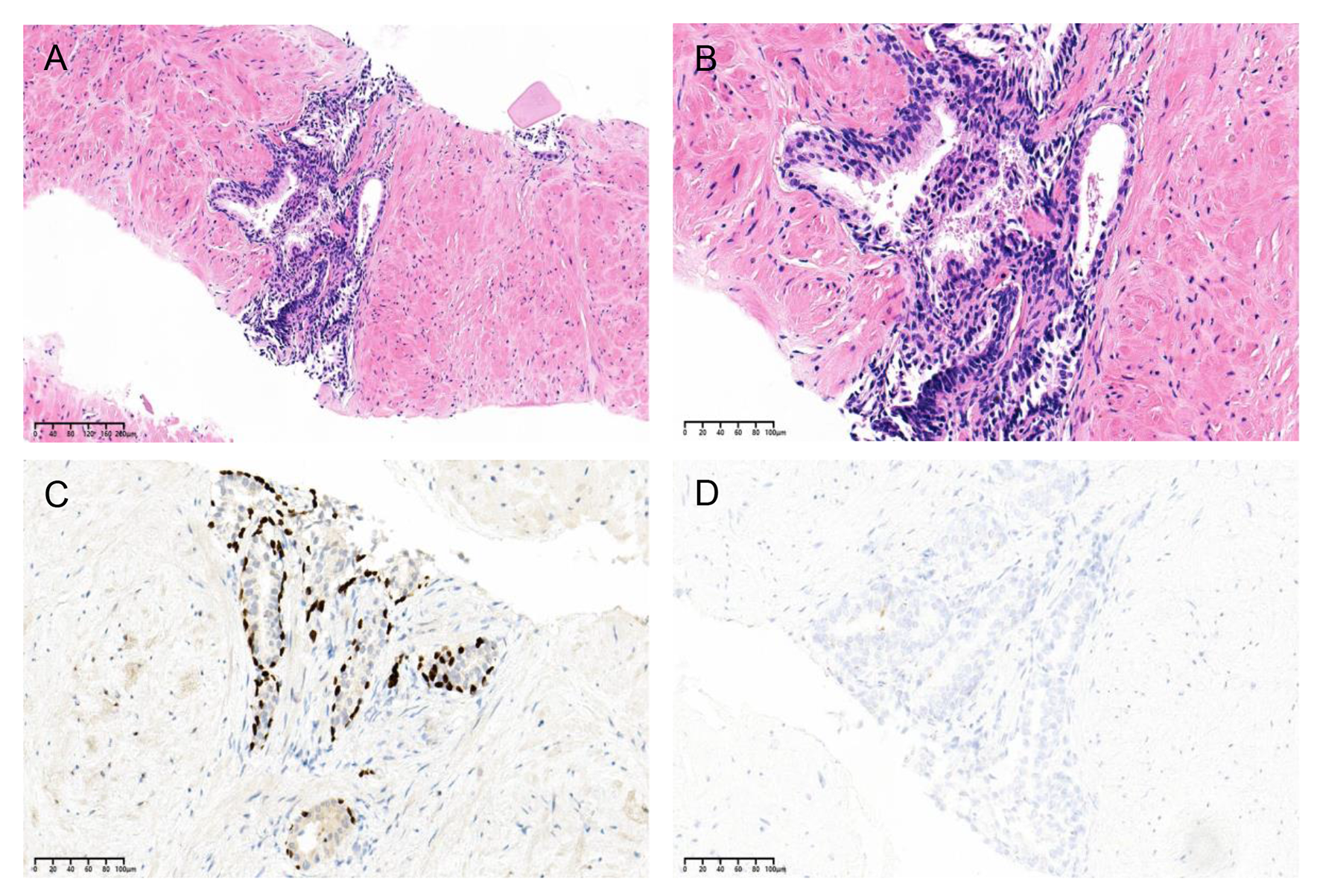

Supplement: Supplementary file 1 [file Image1.tif]
